# Development of a Low-Cost Smart Sensor GNSS System for Real-Time Positioning and Orientation for Floating Offshore Wind Platform

**DOI:** 10.3390/s23020925

**Published:** 2023-01-13

**Authors:** Neus Revert Calabuig, Ismail Laarossi, Antonio Álvarez González, Alejandro Pérez Nuñez, Laura González Pérez, Abraham Casas García-Minguillán

**Affiliations:** Centro Tecnológico de Componentes-CTC, Scientific and Technological Park of Cantabria (PCTCAN), c/Isabel Torres 1, 39011 Santander, Spain

**Keywords:** smart sensor, GNSS system, floating offshore, low-cost sensors, real-time positioning

## Abstract

A low-cost smart sensor GNSS system has been developed to provide accurate real-time position and orientation measurements on a floating offshore wind platform. The approach chosen to offer a viable and reliable solution for this application is based on the use of the well-known advantages of the GNSS system as the main driver for enhancing the accuracy of positioning. For this purpose, the data reported in this work are captured through a GNSS receiver operating over multiple frequency bands (L1, L2, L5) and combining signals from different constellations of navigation satellites (GPS, Galileo, and GLONASS), and they are processed through the precise point positioning (PPP) and real-time kinematic (RTK) techniques. Furthermore, aiming to improve global positioning, the processing unit fuses the results obtained with the data acquired through an inertial measurement unit (IMU), reaching final accuracy of a few centimeters. To validate the system designed and developed in this proposal, three different sets of tests were carried out in a (i) rotary table at the laboratory, (ii) GNSS simulator, and (iii) real conditions in an oceanic buoy at sea. The real-time positioning solution was compared to solutions obtained by post-processing techniques in these three scenarios and similar results were satisfactorily achieved.

## 1. Introduction

Nowadays, the safest and most reliable way to combat the greenhouse effect is the one that goes through renewable energies. The main objective is to replace the current energy model, which is fundamentally based on fossil fuels, with energy from clean and renewable sources to reduce the carbon footprint [1,2]. In this sense, notable advancements are being made in all the renewable energy production forms and particularly in photovoltaic and wind energy, both onshore and offshore [3,4,5,6]. In particular for offshore wind energy, it is vital to take advantage of the potential energy available by locating the turbines in regions with stronger and more consistent winds. Normally, these conditions occur in deep waters. In these scenarios [3], the employment of floating offshore wind turbines (FOWTs) is more suitable and cost-effective in respect to the fixed structures.

Currently, several prototypes or demo projects have been installed worldwide and have demonstrated the viability of different floating structures solutions (e.g., spar, semi-submersible, and tension leg platforms). For instance, the Hywind Scotland Pilot Project, installed in 2017, was the first pre-commercial floating wind park with five large turbines of 6 MW in array formation. Wind Float Atlantic was the second pilot project, with turbines of 8.4 MW—the largest ever installed on a floating platform [7].

The current and forecasted growth of the FOW sector drives the need to develop reliable and dependable station-keeping systems. In this regard, this technology continues to be the subject of importance to deep research aimed at reducing its operating and, mainly, maintenance costs toward improving its economic competitiveness and ensuring a safe working environment. Therefore, these systems must be capable of enduring all damage and degradation mechanisms that can be derived from the environment: corrosion, marine growth, harsh conditions (storms), etc. This requirement leads to getting significant efforts underway to develop more effective and efficient methods of inspecting and monitoring mooring lines to detect early stage signs of degradation and to predict failure. The first aspect is carried out by regular in-service inspections of mooring systems as part of the integrity management program. However, this methodology is generally costly and redundant; offshore inspection requires support vessels, crew, and equipment at a substantial daily rate. In contrast, the monitoring techniques provide the necessary information and the required periodic measurements to assist the structural-health monitoring (SHM) system in the tasks of extracting the fault-sensitive features from the measured signals and using them to analyze the system’s condition [8].

The foremost mooring-line monitoring technologies include sonar probes, inclinometers, load cells, and fiberoptic sensors [9,10,11]. Some authors have also suggested ultrasonic guided waves and acoustic emissions as potential solutions. However, each one of these techniques has serious drawbacks that need to be tackled. The low reliability, accuracy, and robustness, and the limited load range of the load cells or the calibration difficulty to relate angle and mooring-line load in inclinometers or the expensive fiber optic technology are some examples of these disadvantages. Instead, mooring-line failure detection based on positioning systems is an attractive alternative with several advantages, such as (i) being highly cost effective, with minimum hardware and installation costs, (ii) having sensors located in safe areas, and (iii) requiring no wireless transmissions.

The basic idea of this technology is to compare the measured position with the predicted position, which is based both on current meteorology and oceanography conditions, and the position offset is related to line damage [12]. It is important to underlay the key importance of monitoring the floating wind turbine 3D motion in a real-time regime with adequate accuracy, as well as frequency and latency, with a view to assisting a structural-health monitoring (SHM) system. The SHM is focused on detecting and preventing otherwise feasible and unexpected damages. To accomplish these requirements and determine the position with high precision, the global navigation satellite system (GNSS) is commonly used. In this system, a spatial infrastructure allows navigation users, with a compatible receiver, to determine their positions, velocities, and time (PVT) by processing the satellites signals. These signals are provided by constellations of satellites with global (e.g., GPS, GLONASS, GALILEO, and BeiDou) and regional (e.g., QZSS and IRNSS) coverages that are often complemented with satellite-based augmentation systems (e.g., EGNOS) [13].

The determination of the PVT solution in the simplest method can be implemented with a GNSS antenna and receiver that capture the data and send them to a processing unit that provides the solution. This technique is easy to use and fast to compute; however, the accuracy obtained through this positioning technique is usually around 10 m, which is not enough in some demanding applications. To overcome this limitation, differential or relative positioning techniques (e.g., GNSS differential or real-time kinematics (RTK)) are mainly used. In their implementation, at least two receivers and antennas are employed in different positions. One of them acts as the base station and, generally, its position is known with high accuracy. Both receivers track the same satellite signals and the relative position coordinates of the rover are determined. The main drawback of this implementation is the accuracy dependence with the baseline length that separates the base and rover stations. In the RTK case, the maximum baseline length is limited to a short-range distance (30–50 km), since in this area the satellite clock and the atmospheric conditions are similar in both receivers and errors can be successfully removed [14,15].

Another promising solution used to improve the positioning accuracy is called the precise point positioning (PPP) technique, in which the absolute and differential positioning algorithms are combined [16,17]. Unlike the RTK solution, the PPP algorithm does not require the existence of a nearby base station but instead needs pseudo-range and carrier phase measurements, as well as the precise knowledge of the satellites’ clock offsets and orbits. These last data are provided by external organizations in real time (usually involving a cost) or in final product form, available after a period of time (usually 15 days) [18]. Some private companies have developed a worldwide network infrastructure to generate the needed data and serve them via satellite communication; however, this approach cannot be implemented by all the applications because of the high costs and the need to employ specific hardware [19,20]. Another alternative is computing the PPP solution from the open-access real-time corrections provided by the International GNSS Service (IGS) following the RTCM (Radio Technical Commission for Maritime Services) standard via NTRIP (networked transport of RTCM via internet protocol) [19,20,21]. The principal disadvantage of this technique lies in the long convergence time (between 20 min and some hours) to achieve centimeter-accuracy solutions, a factor that limits its use in some real-time applications. To minimize this drawback, multi-constellation and multi-frequency receivers are normally employed. In this way, it is possible not only to reduce the convergence time but also to allow for better ionosphere errors resolution (thanks to ionosphere-free combinations) and, therefore, more successful resolution of ambiguities, which results in a highly accurate position solution. Consequently, the use of the PPP technique and multi-frequency receivers provide an advantageous option for numerous commercial applications, such as precision agriculture, geodetic surveying, airborne mapping, etc., where the operability is unaffected by initial convergence periods. In the specific case of offshore implementations, especially scenarios on high sea (>50 km from the coast) installations, where the RTK algorithm is not suitable, the PPP technique is the unique solution and, currently, there is intense research to improve its performance [19,22]. To facilitate the PPP solution computation, the IGS service provides different precise products; among them are the IGS ultra-rapid products (IGU) and the multi-GNSS experiment (MGEX). The IGU products are the IGS predicted products that are available for real-time and near real-time use. The IGU products are only available for GPS and GLONASS. The MGEX has been set up by the IGS for tracking and analyzing all available GNSS signals and any space-based augmentation system (e.g., SBAS); these products are available after some days or weeks [23,24,25].

GNSS solutions, and especially techniques such as PPP and RTK, provide precise position and velocity information. However, GNSS methods also have some weaknesses. They typically provide low data output rates (typically 1–10 Hz), and signals are vulnerable to jamming (even unintentional interference), multipath, receiver noise in low-cost receivers, etc. For this reason, usually, other technologies are used to complement this algorithm and ensure constantly accurate position measurements even when GNSS data are unreliable [26,27,28]. Among the integration approaches, the inertial navigation system (INS) is a promising solution because of it being cost-effective and not requiring additional infrastructure. This system uses rotation and acceleration information from an inertial measurement unit (IMU) to calculate relative position over time. Once this last parameter is estimated, it is fused with the GNSS measurements to improve the accuracy and to serve positioning data with high frequency [29].

In this paper, the results obtained to validate the developed low-cost smart sensor GNSS system for real-time positioning and orientation for floating offshore wind platforms are presented. The proposed system takes advantage of the GNSS system and uses innovative techniques that combine multi-constellation and multi-frequency. Furthermore, for a high and accurate positioning, the GNSS solution is fused with the data acquired from an IMU, reaching final resolutions of 20 cm. Furthermore, besides the precision (<20 cm), the developed system has to comply with some technical restrictions such as velocity accuracy < 3 mm/s, attitude accuracy < 0.2 deg, data output rate of at least 1 Hz, a delay in the real-time output of 0.5 s, and a maximum bandwidth of 50 Kbps. The total cost of the Smart Sensor system can be around EUR 3000 and may vary slightly depending on the components included (receiver, antenna, inertial measurement unit, etc.). This represents a cost savings of approximately 90%, over a 25-year lifespan, in comparison with other monitoring technologies. When the smart sensor technology reaches high technology readiness levels (industrialization), a medium-scaled batch could save an additional 10–20% in material costs [30].

The validation of the smart motion sensor proposed was accomplished following a step-wise approach, starting from simple algorithm testing and verification of basic functionalities, and incrementally adding complexity to better represent real operation conditions. For this reason, three sets of tests were carried out: first, in a rotary table at the laboratory to check the sensor performance in the early stages of its development. After that, the registered motion of a floating platform in a water tank was used as input into the GNSS simulator, emulating similar conditions in platform locations. Finally, the low-cost smart sensor was validated in a real sea scenario. It should be noted that the development has been under certain requirements and restrictions such as low-cost perspective, modularity, and safety, as will be detailed in the following sections.

The development of this smart sensor is part of the European project called MooringSense (https://www.mooringsense.eu/, accessed on 12 December 2022), whose main objective is to develop a digital twin that reduces operational costs (minimizing premature failure rates and inspection costs and assisting in scheduling maintenance operations) and, at the same time, increases efficiency by developing an efficient risk-based integrity management strategy for mooring systems based on affordable and reliable online monitoring technology.

The remainder of the paper is organized as follows: Section 2 elaborates a summary of the materials employed to assemble the smart sensor developed, the main dataflow diagrams, the block system sketch design, hardware, and a methodology road map up to the completion of the test campaign. Section 3 is aimed at summarizing the test results obtained for the static mode, the ocean basin site, the GNSS motion simulation, and into the real scenario (sea laboratory BIMEP). Last, but not least, Section 4 concludes all observations gathered and discussed in previous sections.

## 2. Materials and Methods

### 2.1. Materials

**Figure 1:**
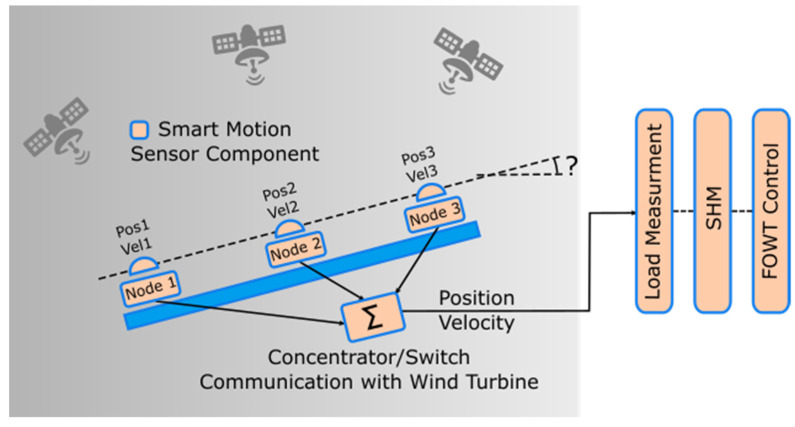
Mooring sense smart sensor system block.

As already mentioned, the main objective of the smart sensor is to monitor the floating wind turbine’s 3D motion and register it in real time with the required accuracy, frequency, and latency to be usable by other modules in the platform system. The registered data help the digital twin in the estimation of loads at the mooring lines and, therefore, enables the SHM module to detect unexpected damage and use it as feedback to the closed-loop control algorithms to reduce loads. For that, the baseline design proposed was the strategic placement of three smart sensors on the platform and a concentrator unit that is responsible for receiving the data from each sensor, processing these data, packing them together, and delivering them to the digital twin. Figure 1 shows a block diagram of the proposed design.

**Figure 2:**
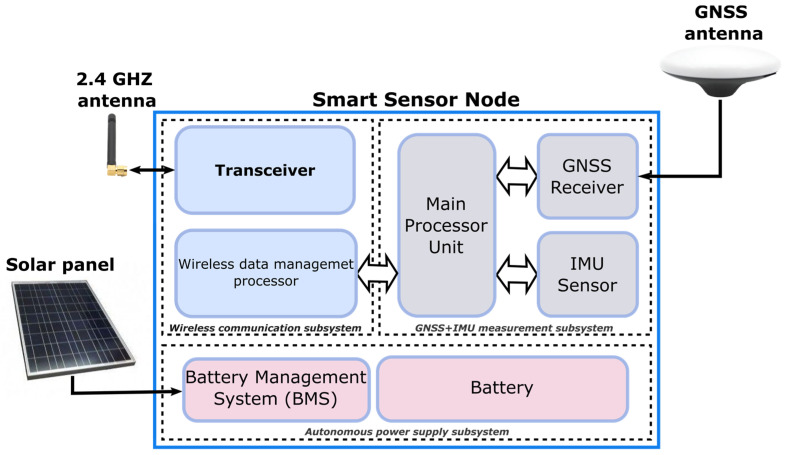
Smart sensor node block diagram.

At the hardware level, each smart sensor node consists of three main blocks: (i) GNSS receiver and antennas, (ii) IMU, and (iii) processing unit. As the sensor must be based on cutting-edge mass-market technology, the GNSS receiver selected is an Ublox ZED F9P, a multi-band GNSS module with integrated multi-band RTK technology for centimeter-level accuracy. The inertial measurement unit chosen is the Xsens MTi-610, which is an industrial-grade, affordable MEMS-based orientation sensor with an easy-to-use open SDK [31]. It is a fully functional, self-contained module that contains a three-axis gyroscope, three-axis accelerometer, three-axis magnetometer, barometer, high-accuracy crystal, and low-power microcontroller unit. The main processing unit is based on the Raspberry Pi3 Model B+ because of its elevated efficiency, since it combines high computational power with low consumption, which makes it ideal for the concerned application. Furthermore, the system is equipped with two antennas: the first one is dedicated to GNSS signal reception (Tallysman TW3972, Kanata, ON, Canada), which is able to receive signals from multi-constellations and multi-frequencies, and its IP67 certification makes it suitable for maritime environments, while the second one is used for the wireless communication (@2.4 GHz) between smart sensor nodes and the concentrator unit and for receiving the real-time corrections. Finally, concerning the power supply of each node, a solar panel (SOLARA S320P41, Hamburg, Germany) is included to generate direct current and charge the battery integrated inside the smart sensor through the autonomous power supply subsystem. In this sense, a battery of 300 Wh and its corresponding power management system were selected to provide the system autonomy for more than 3 days even without any solar charge. The detailed block diagram is presented in Figure 2.

**Figure 3:**
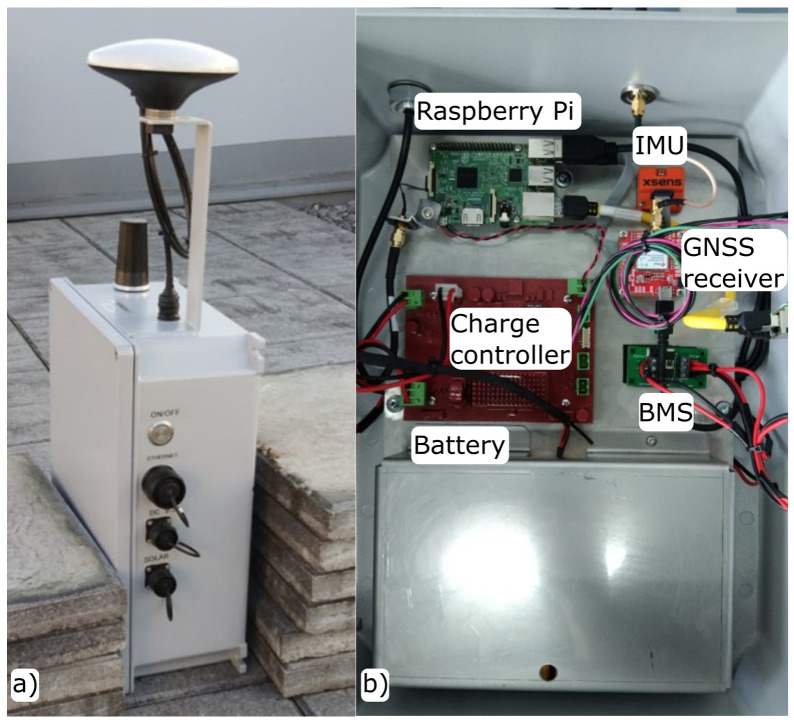
Smart sensor (**a**) waterproof housing and (**b**) internal view.

Figure 3a presents the design proposed integrated inside a waterproof housing (machined to install the connectors needed) that corresponds to the international standard rating of IP68, withstanding dust, dirt, and sand and providing the necessary resistance to submersion up to a maximum depth of 1.5 m underwater, which makes it appropriate for the environment. For its part, in Figure 3b, the smart sensor components and placement inside the said enclosure are depicted.

**Figure 4:**
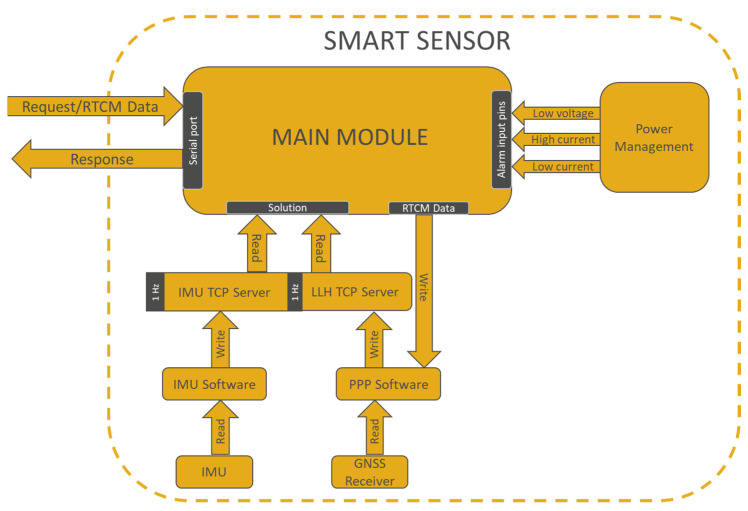
Smart sensor data flow.

Figure 4 shows the software architecture of the smart sensor. The software has been developed under a Raspbian operating system. It manages the proper behavior and the correct communication between all parts that integrate it, in such a way that it sends the precise 3D motion to the concentrator. The main module oversees the coordination of the different parts of the system, handling its requests and responses and delivering the position or attitude solution to the concentrator when requested. It is also in charge of checking the sensors and power system alarms and reporting them and receiving the RTCM data that will be used by the real-time PPP software (RTKlib) for positioning estimation.

The PPP software runs in real time using data obtained from the GNSS receiver and RTCM, respectively, to compute the precise positioning of the platform. At each system startup, it creates a TCP server and, once the required precision has been achieved, publishes the solution at a fixed frequency of 1 Hz. The IMU software, for its part, receives accelerometer, gyroscope, and magnetometer readings to calculate its attitude and publishes it on another TCP server to be available to any client. The interconnection between the main processor unit, the GNSS receiver, and the IMU sensor is provided through an UART port.

### 2.2. Methods

In this section, the different levels of testing realized in this work will be explained, detailing the different setups and plans for each one. The tests are carried out under different scenarios to validate the functionality of the smart sensor and confirm compliance with the previous specifications imposed by the application. In this sense, four different sets of tests were accomplished:

▪ Fixed antenna test (static and kinematic mode processing);
▪ Test in ocean basin using GNSS simulator (general receiver functionality);
▪ Tests in motion simulator (kinematic mode);
▪ Tests in sea scenario (kinematic mode).

**Figure 5:**
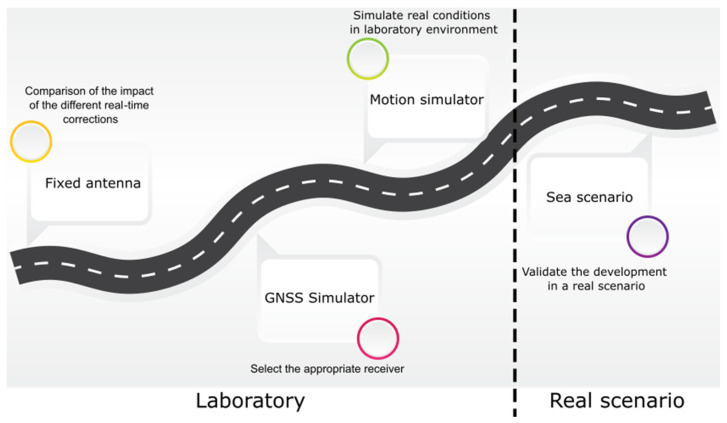
Road map followed in the smart sensor tests.

These tests have followed a road map with increasing difficulty, validating at each stage the parts and aspects that influence the final solution of the system. Figure 5 shows a diagram of this methodology.

#### 2.2.1. Fixed Antenna

According to the planned methodology, a stepwise approach was followed to validate the proposed and developed smart sensor. In that respect, the first stage was to verify the validity of the suggested system and the performance of the proposed algorithms. Several tests in static and kinematic modes were implemented to compare the solution accuracy obtained in both modes. This set of tests was also useful to compare the impact of the different real-time corrections in the final solution and choose the best one for our purpose.

#### 2.2.2. Tests in Ocean Basin with GNSS Simulator

Between August and October 2021, tests were carried out with a scaled-down physical model of the SAITEC-SATH floating offshore wind turbine in the ocean basin (https://www.sintef.no/en/all-laboratories/ocean-laboratory/, accessed on 10 December 2022) at the SINTEF ocean facilities. A model scale of 1:36 was used, where the expected environmental condition of the FOWT were reproduced. The test conditions were based on the Buchan deep-site environment.

In these tests, the R&S SMBV100B GNSS signal simulator was employed to provide artificial GNSS data from GPS, Glonass, Galileo, BeiDou, and QZSS. It works in a large range of frequencies (from 8 KHz to 6 GHz), covering all the important RF bands for digital wireless communications. The instrument is useful for the computation of multi-constellation and multi-frequency solutions at different scenario configurations and dynamics modes.

Using the same smart sensor used in the tests in the previous section but without the antenna, since the GNSS data were supplied by the simulator, providing the precise position for both the rover and base station (5 km distance), the test plan conducted was divided into static and kinematic tests. In static mode, the rover position was the technological center CTC location (43.921, −3.9552, 113), while in kinematic mode, datasets from simulation tests were utilized to replicate the dynamics on the floating platform. Unlike the previous case, the output RINEX OBS and NAV files were post-processed by the RTK algorithm. The different cases used for the tests are exposed in Table 1, where it can be noticed clearly that different waves and inclinations were considered. The main objective of these tests was to select the appropriate receiver and validate it in a simulation scenario.

#### 2.2.3. Tests in Motion Simulator

**Figure 6:**
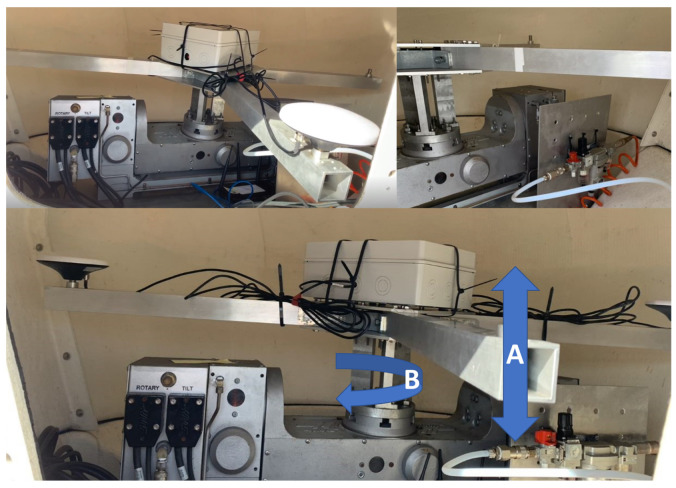
Motion simulator. Vertical (A) and horizontal (B) axis.

The motion simulator is a two-axis rotary table that is a fully automatic programmable positioning device that can be moved to different positions, permitting versatile configurations. The rotary table used is the Haas TRT tilting and it is located inside a radome installed on the roof of the CTC building, which allows a good GNSS signals reception, protecting the system under possible bad climatic conditions. It can provide different movements, combining motions around the vertical (A axis) and the horizontal (B axis) axes, as shown in Figure 6.

To validate the smart sensor performance in this scenario, three different movements were created: rotation around (1) A axis, (2) B axis, and (3) around the A and B axes simultaneously. In the rotary table, a cross brace was placed where the antenna was situated.

**Figure 7:**
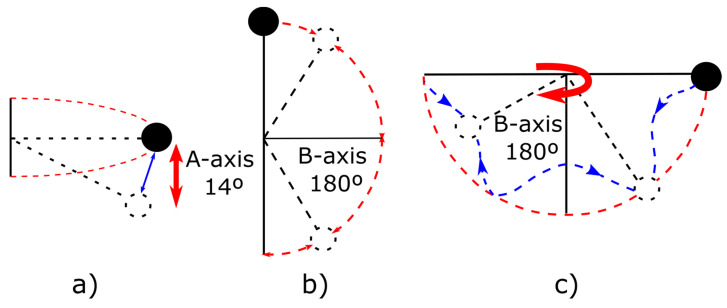
Motion around: (**a**) A, (**b**) B, and (**c**) A and B axes simultaneously.

Due to the small space inside the radome, the movement in the A-axis was limited between 0 and 14° and repetitive movements between 0 and 180° were programmed in the B-axis, as detailed in Figure 7. The black point represents the antenna at the start and changes color to white when the rotary table starts moving (the antenna changes its position). Finally, the last test was carried out mixing both movements to simulate the platform motion. All these tests were performed in slow motion, changing 1° every second.

#### 2.2.4. Tests in Sea Scenario

**Figure 8:**
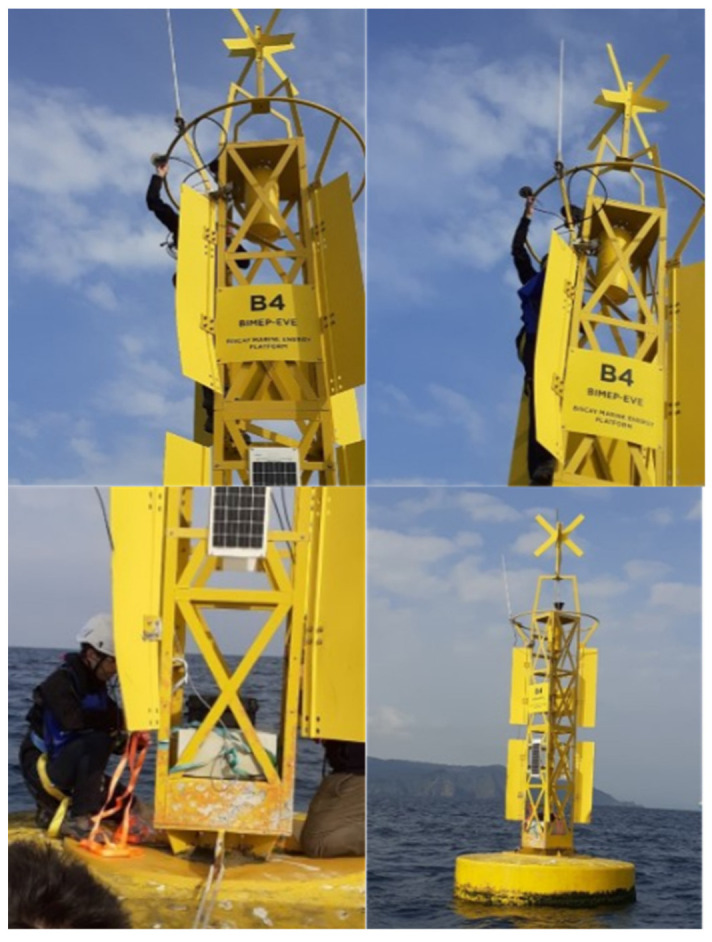
Smart sensor installation on the marking buoy.

The tests in the sea scenario have been accomplished in two steps. First, to check the proper functioning of the proposed approach (Figure 1) two smart sensors and one concentrator were installed in a floating platform (HarshLab (https://www.bimep.com/harshlab/, accessed on 14 November 2022)). With this setup, the first measurements were accomplished in a port environment in view of validating and adjusting the whole system, in terms of validation of the final solution, communication between the nodes themself and with the concentrator and energy consumption. Second, to verify the real-time positioning, one smart sensor was installed in a perimetral marking buoy. The objective here is to validate the smart sensor design and adjust the real-time PPP algorithm in a real scenario, to comply with the application specifications and properly register the platform motions. Accordingly, some modifications were carried out in the node design to optimize the system to include as many power supplies as possible and a 4G access point to monitor in real time the solution data. These tests have taken place at perimetral marking buoy for 15 days. Figure 8 shows the pictures of the smart sensor installation on the marking buoy.

## 3. Results

In this section, the results of all performed tests with the proposed and developed smart sensor in the different above-mentioned scenarios will be presented and analyzed, following the same structure as presented in the previous section.

### 3.1. Tests in Static Mode

**Figure 9:**
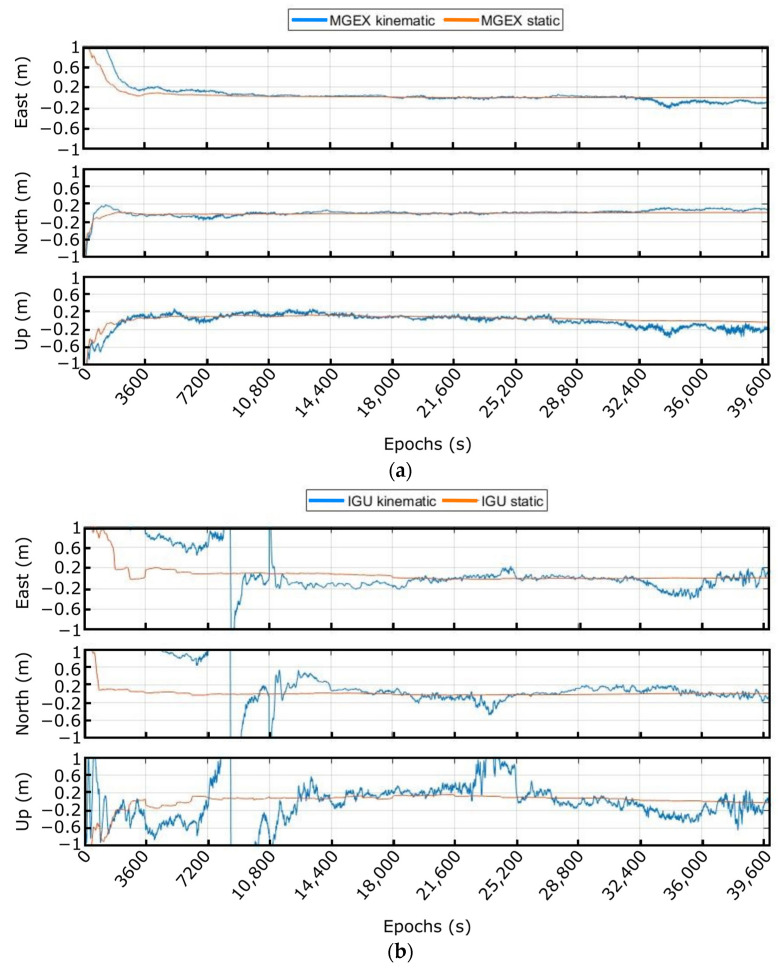
Post-processing results using (**a**) MGEX and (**b**) IGU precise products.

For the static test, the smart sensor was placed on several occasions (to cover different configurations and sky conditions) inside the radome and arranged to use multi-constellation and multi-frequency configurations to compute the real-time PPP solution. First, once the smart sensor was mounted, measurements with post-processing techniques (using the PPP technique and MGEX and IGU precise products), keeping the antenna static, with different precise products (International GNSS Service ultra-rapid products, IGU and multi-GNSS-exchange (MGEX) format) were carried out to determine which one provides the better solution with the selected hardware (HW) components. These tests were useful also to characterize the customized algorithm in terms of convergence time and accuracy. Figure 9 presents a fragment of the obtained results in kinematic (receiver and antenna in fixed position but processing the results in movement) and static (keeping the antenna in a fixed position) modes with both precise products in east, north, and up (ENU) components. In the X axis, the time is presented in epochs and each square (3600 epochs) corresponds to 1 h.

It can be noted that the convergence time with the MGEX precise products is around 30 min and 1 h in static and kinematic modes, respectively, and that the accuracy was kept below ±20 cm in both cases. With IGU’s precise products by their side, the achieved convergence time in static mode has risen to almost 1 h, whilst in kinematic mode this time has exceeded 4 h. On the other hand, the acquired accuracy in static mode was similar to the previous one. However, in kinematic mode, in the east and north component, the requirement to keep the error below ±20 cm was fulfilled; nevertheless, in the up component and after the convergence period, the error exceeds the allowed. Therefore, it can be concluded that the quality and the type of products for clocks and orbits influence the results.

**Figure 10:**
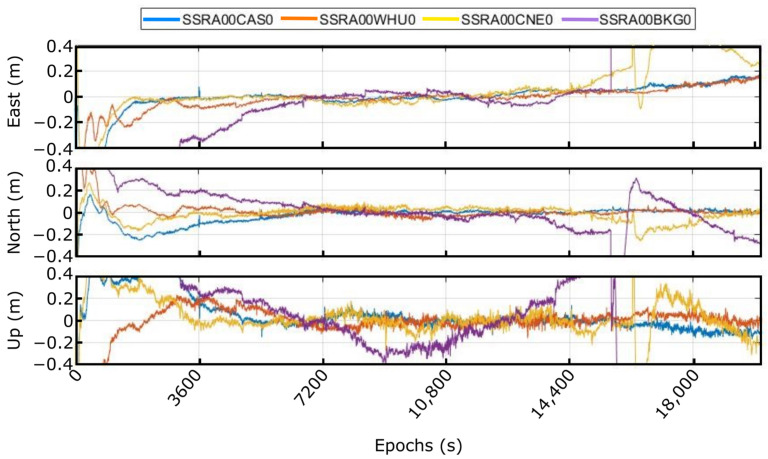
Real-time solution with different orbits and clock corrections in kinematic mode.

As a second set of tests in this case, PPP real-time solutions were computed with different real-time corrections to analyze their impact on the quality of the results. These real-time corrections are provided by several NTRIP broadcaster providers, and the precise orbits and clocks needed by the algorithm can be derived from them with periodicity of 60 s and 10 s, respectively. Figure 10 shows in ENU components, a capture comparing four different real-time corrections (SSRA00WHU0, SSRA00CAS0, SSRA00CNE0, and SSRA00BKG0).

As can be clearly seen, the quality and continuous availability of these corrections impact directly on the final solution. For instance, the corrections SSRA00CNE0 and SSRA00BKG0 present the worst results, with high discontinuity jumps around the 4th and 5th hour, while the other two show more stability, keeping throughout the measurement time the error below the specified. These worst results could be due to the type of receiver and antenna used or the quality of the signal and, also, due to multipath or problems when receiving the signal.

### 3.2. Tests in Ocean Basin with GNSS Simulator

**Figure 11:**
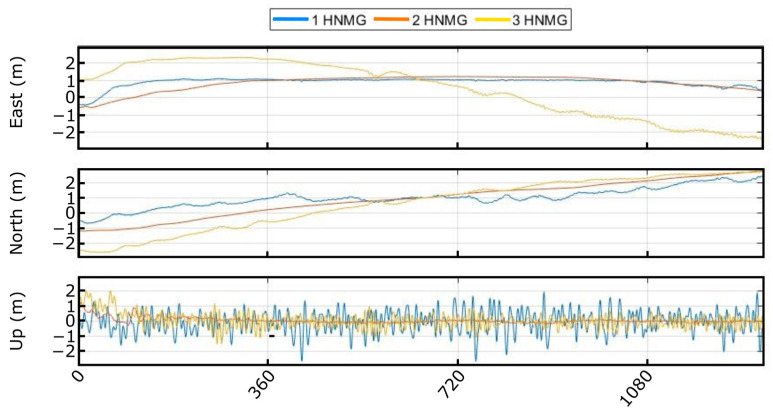
Different solutions with the GNSS simulator.

The main objective to use the GNSS signal simulator was to validate the receiver performance under conditions similar to those that can be found on the deep sea, generating GNSS RF signals such as the ones obtained with a GNSS antenna in real operations. Figure 11 presents the different solutions achieved employing the GNSS simulator for 20-min captures, where solutions with different turbulences and wind speeds are compared. As indicated in Table 1, three conditions were emulated, and the output generated was post-processed to obtain the RTK solution.

▪ 1 HNMG: this simulation presents extreme model turbulence, high wave height (10.5 m), and high wind speed (25 m/s). Due to these conditions, the up component presents tall height variations, and the displacement in the horizontal domain (east and north components) are not especially relevant.
▪ 2 HNMG: in this case, a typical situation is emulated with the lower wind speed (4 m/s), normal turbulence, and low wave height (1 m). In this solution, the displacement is the lowest and in the up component varies slowly.
▪ 3 HNMG: this simulation presents high wind speed (20 m/s), normal turbulence, and low wave height (3.8 m). Due to the high wind speed and the lower turbulence, the displacement is higher in the horizontal domain (east and north).

**Figure 12:**
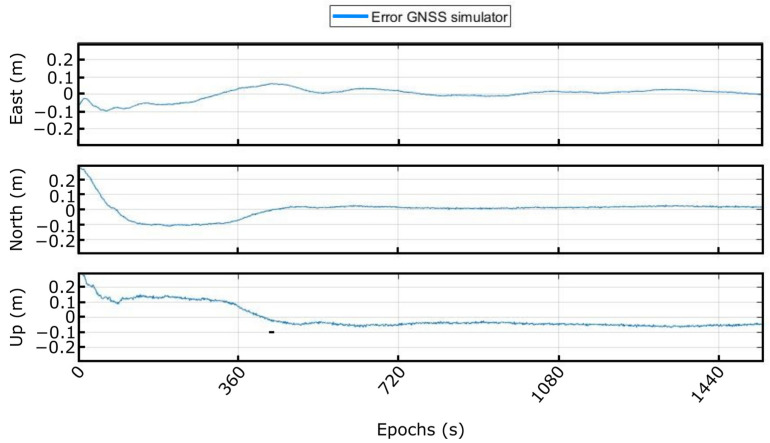
Solution error achieved in the GNSS simulator.

To compute the position error, this final solution has been compared with the files with the movement provided to the GNSS signal generator. Figure 12 presents the error achieved when using the GNSS simulator and post-processing technique. As expected, the RTK positioning error is below ±10 cm once the solution has converged.

### 3.3. Tests in Motion Simulator

In this section, different results from the afore-named motion simulator will be presented. Following the steps described in Section 2.2.2, the validation of the smart sensor and the real-time PPP algorithm in the three tests (movement around A, B, and A and B axes simultaneously) will be discussed.

**Figure 13:**
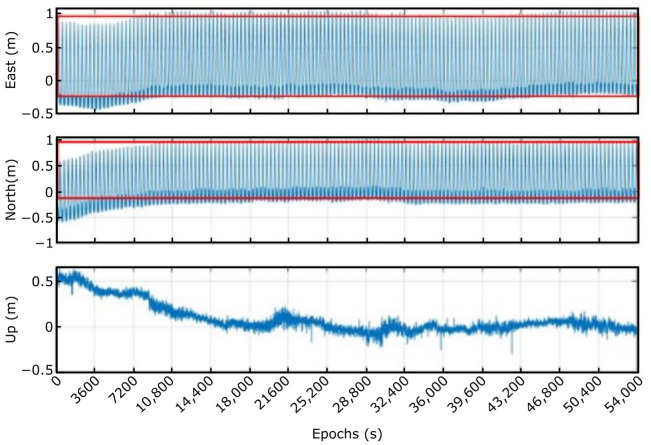
Solution rotating around B axis.

The results achieved with the first test that was carried out performing periodic movements around the B axis (±180°) are exhibited as ENU components in Figure 13. The results, as can be observed, represent, approximately, a half sinusoidal movement accordingly to the plan. The small peaks that appear in the east and north components occur because the axis in the rotation table is relative to its reference position and not to the ENU components. The red squares in the figure indicate where the solution values exceed the programmed movements. As can be seen, after some steps, the solution converges, and the results are within the average.

**Figure 14:**
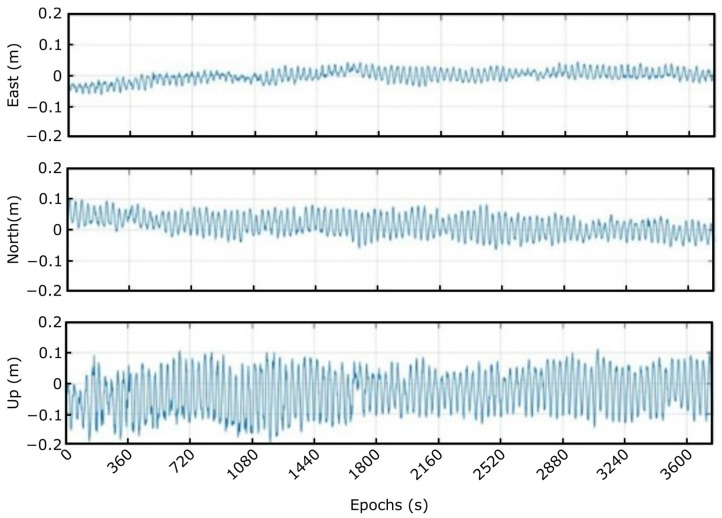
Solution rotating around A axis.

The other test conducted in the motion simulator was relative to the rotation around the A axis. As previously mentioned, due to space problems inside the radome, these movements were limited to 0–14°. The results obtained in this test are not obvious as those tested before. Ideally, only the up component must change (about 17 cm as shown in Figure 14) with the movements, and the other two should remain constant. However, these last two components also show small variations but always within the established margin of error (20 cm). These small errors may be caused by the vibrations produced in the movement of the platform and by the error of the real-time PPP solution itself.

**Figure 15:**
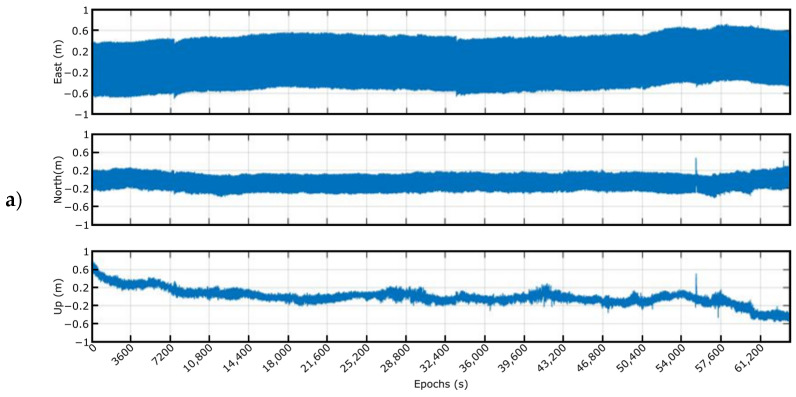

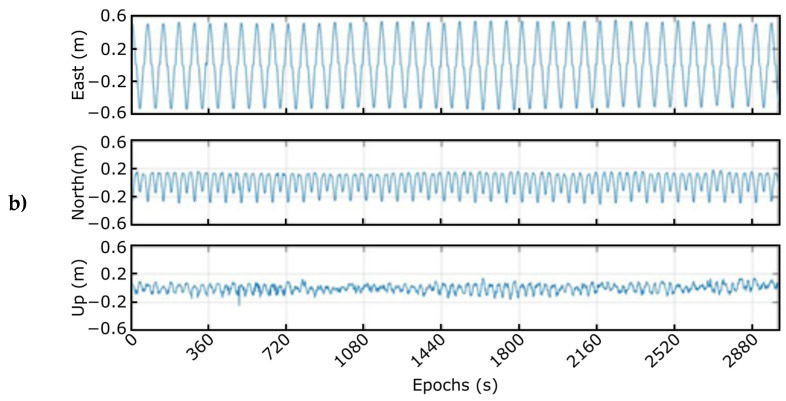
Solution rotating around A and B simultaneously: (**a**) 18 h, (**b**) 1 h.

The last test in the rotary table was the movement combination of both axes to simulate the 3D platform motion. A capture of the results of this test that was taken around 18 h is represented in Figure 15. Observing Figure 15a, it can be noticed that the system is able to maintain the same dynamics during a long data capture. It is important to remark that in the case of losing convergence, the algorithm is able to recover it quickly, as can be distinguished around the 7200 and 54,000 epochs. To show these movements more clearly, in Figure 15b, one hour of the calculated solution is displayed. The movement’s periodicity in the east and north components is distinctly noted. The up component, however, shows small variation because of the high sensibility of this component with the geometry of the satellites.

### 3.4. Tests in Sea Scenario

**Figure 16:**
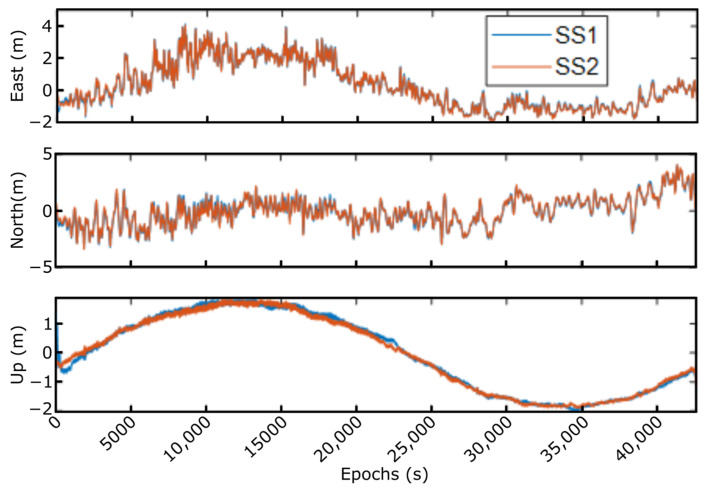
Test results in the HarshLab platform at the port.

According to the test plan detailed in Section 2.2.4, two sets of tests were carried out. The first measurements were accomplished in the port environment to check the proper functioning of the system installed (the two smart sensors and the concentrator) in the HarshLab platform. As an illustrative example of the correct behavior of both smart sensors (SS1: Smart Sensor 1 and SS2: Smart Sensor 2), Figure 16 shows a period of results obtained in the verifications tasks.

**Figure 17:**
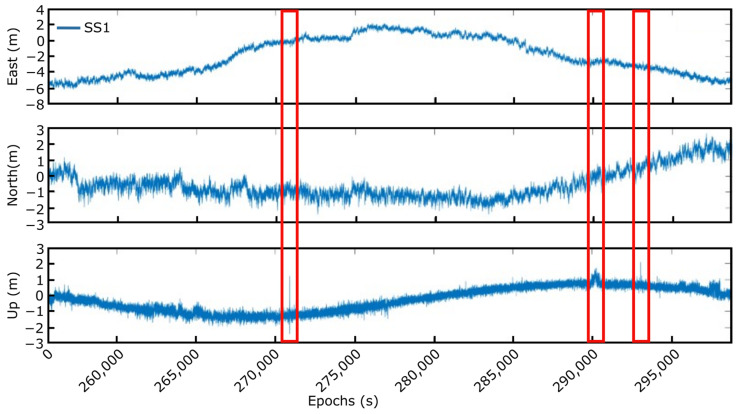
Twelve-hour measurement at the marking buoy.

As mentioned before, the second set of tests was performed by only one smart sensor on a marking buoy in the Bimep area (https://www.bimep.com/, accessed on 14 November 2022), with some optimizations that were introduced in the system, especially in the employed battery, to increase the duration of the measurements. The main drawback with this new configuration was the determination of the solution’s accuracy. Using only one smart sensor and the low frequency of the data provided by the oceanographic buoy notably hinder the validation process of the proposed approach. In this sense, the precision of the solution provided by the smart sensor was calculated as the difference between this data and the results achieved by applying the RTK technique with the base station closest to the Bimep area. Considering the previous results obtained with the smart sensor and RTK post-processing technique, it is shown that the error is always less than 10 cm, and it can be considered that this method can be used as a precise reference solution (as the real position of the buoy). Therefore, and considering all the above-mentioned, an interval of the smart sensor data obtained during the real-time tests is shown in Figure 17. In these measurements, the sensor was able to provide, with some punctual losses of corrections, the estimated position of the buoy continuously and with the established precision, as will be discussed later. In the moments that the corrections were lost (red rectangles in the figure), the sensor is capable of rapidly converging and recovering normal operation, providing the real positioning measurements.

**Figure 18:**
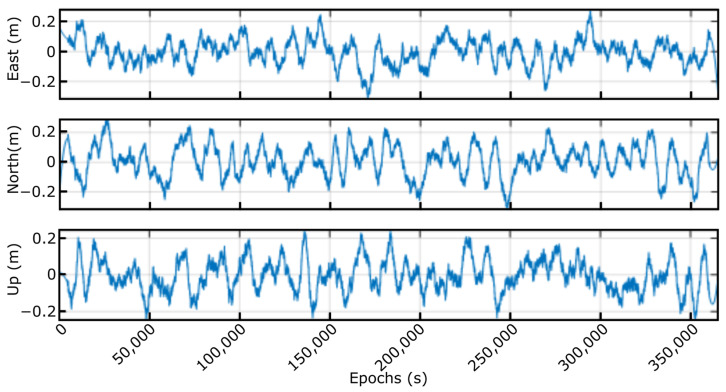
Error in east, north, and up components.

The Figure 18 shows the error achieved in ENU components, calculated as the difference between the smart sensor solution and the one obtained by applying the RTK algorithm to raw captured data. As can be clearly noticed, the error was always below the pre-established value (+/−20 cm), except when the corrections were lost eventually, where the system is capable of reconverging rapidly and quickly, providing the correct solution.

## 4. Conclusions

This work arises from the continuing need to reduce operational costs and, at the same time, increase floating offshore efficiency. The smart sensor forms part of the MooringSense project that aims to achieve these goals, developing an efficient risk-based integrity management strategy for mooring systems based on affordable and reliable online monitoring technology. Several tests at different scales were carried out to verify the good performance and suitability of the smart sensor and the algorithms developed. These tests verify its viability for use in real-time monitoring of offshore platforms. All the measurements carried out, starting from the static mode to the validation in the real environment, have shown that the developed system successfully complies with the specifications imposed by the application: positioning precision < 20 cm, velocity accuracy < 3 mm/s, attitude accuracy < 0.2 deg, data output rate of at least 1 Hz, a delay in the real-time output of 0.5 s, and a maximum bandwidth of 50 Kbps. In this sense, the preliminary tests in static mode have provided the possibility to choose the appropriate corrections for future use in real time in real scenarios. Following the strategy of using low-cost components, the tests with the GNSS simulator have allowed the selection and validation of the lowest-cost receiver suitable for the application. Increasing the difficulty and complexity, tests have been carried out in the motion simulator with real wave movements to validate the developed system at the laboratory level. Finally, once the correct functioning of the smart sensor has been verified, tests have been carried out in a real environment. First, in a controlled environment (the port) to verify, on the one hand, the correct integration and operation of the different parts of the smart sensor and, on the other, between the different nodes (two in this case) and the concentrator. Later, its correct behavior in the open deep sea has been verified by installing it on a buoy.

In all of them, satisfactory results have been obtained, determining the position (both in real time and in post-processing) with precisions below 20 cm, the principal objective of this development, which will help the other tools (mainly the digital twin) to optimize the performance of these platforms and substantially reduce their costs of production and maintenance. Analyzing several parameters that affect directly or indirectly the behavior of the sensor, such as all HW components used (separately and as a whole system), the algorithms, the environment (multipath and interference), the corrections, and all those that can interfere with the accuracy of the measurement, it was possible to design a GNSS system monitoring with very low cost and high performance. Finally, it should be noted that although the designed prototype corresponds to low technology readiness levels, it has shown great robustness and reliability. By improving some features such as convergence time and point solution losses, it can be taken to higher levels and provide a very safe and low-cost commercial alternative.

## Figures and Tables

**Table 1:** Different load cases selected to compute the solution.

Simulation ID	DLC (Design Load Cases)	Mean Wind Speed at Hub Height (m/s)	Wind Direction (deg)	Turbulence Model and Intensity	Sea Speed (m/s)	Significant Wave Height (m)	Peak Period (m)
1 HNMG	6.1	25.0	330.0	Extreme—0.11	0.41	10.5	14.3
2 HNMG	6.4	4.0	330.0	Normal—0.26	0.41	1.0	6.0
3 HNMG	6.4	20.0	330.0	Normal—0.12	0.41	3.8	9.1

## Data Availability

Not applicable.
